# Electronically monitored occlusion therapy in amblyopia with eccentric fixation

**DOI:** 10.1007/s00417-021-05416-5

**Published:** 2021-10-16

**Authors:** Berna Mehmed, Maria Fronius, Tabea Pohl, Hanns Ackermann, Charlotte Schramm, Bettina Spieth, Christian Hofmann, Thomas Kohnen, Yaroslava Wenner

**Affiliations:** 1 Department of Ophthalmology, Goethe University Hospital, Frankfurt am Main, Germany; 2 Department of Biostatistics and Mathematical Modelling, Goethe University Hospital, Frankfurt am Main, Germany; 3 Department of Ophthalmology, Eberhard Karl University Hospital, Tübingen, Germany

**Keywords:** Amblyopia, Eccentric fixation, Occlusion treatment, Dose response, Efficiency

## Abstract

**Purpose:**

Amblyopia with eccentric fixation, especially when not diagnosed early, is a therapeutic challenge, as visual outcome is known to be poorer than in amblyopia with central fixation. Consequently, treatment after late diagnosis is often denied. Electronic monitoring of occlusion provides us the chance to gain first focussed insight into age-dependent dose response and treatment efficiency, as well as the shift of fixation in this rare group of paediatric patients.

**Methods:**

In our prospective pilot study, we examined amblyopes with eccentric fixation during 12 months of occlusion treatment. We evaluated their visual acuity, recorded patching duration using a TheraMon®-microsensor, and determined their fixation with a direct ophthalmoscope. Dose-response relationship and treatment efficiency were calculated.

**Results:**

The study included 12 participants with strabismic and combined amblyopia aged 2.9–12.4 years (mean 6.5). Median prescription of occlusion was 7.7 h/day (range 6.6–9.9) and median daily received occlusion was 5.2 h/day (range 0.7–9.7). At study end, median acuity gain was 0.6 log units (range 0–1.6) and residual interocular visual acuity difference (IOVAD) 0.3 log units (range 0–1.8). There was neither significant acuity gain nor reduction in IOVAD after the 6th month of treatment. Children younger than 4 years showed best response with lowest residual IOVAD at study end. Efficiency calculation showed an acuity gain of approximately one line from 100 h of patching in the first 2 months and half a line after 6 months. There was a significant decline of treatment efficiency with age (*p* = 0.01). Foveolar fixation was achieved after median 3 months (range 1–6). Three patients (> 6 years) did not gain central fixation.

**Conclusion:**

Eccentric fixation is a challenge to therapy success. Based on electronic monitoring, our study quantified for the first time the reduction of treatment efficiency with increasing age in amblyopes with eccentric fixation. Despite some improvement in patients up to 8 years, older patients showed significantly lower treatment efficiency. In younger patients with good adherence, despite poor initial acuity, central fixation and low residual IOVAD could be attained after median 3 months. Hence, the necessity of early diagnosis and intensive occlusion should be emphasized.

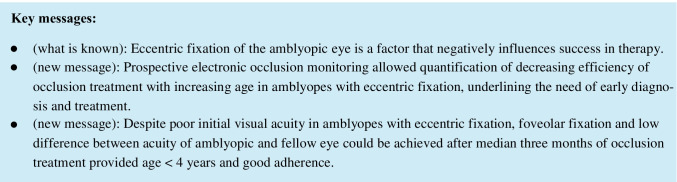

## Introduction

Amblyopia is the most common vision abnormality among children and is a significant cause of a lifelong deficiency of visual acuity. It often affects a single eye and is characterized by a decreased best corrected visual acuity for which no organic lesion can be detected [1]. Any asymmetry in the quality of visual input such as in anisometropia and/or strabismus or deprivation during the sensitive period leads to abnormal development of the binocular visual system [2]. In some rare cases, this results in the development of an eccentric fixation [3]. The amblyopic eye is unable to fixate with the foveola when the fellow eye is covered. Eccentric fixation can be diagnosed after the age of 4–5 months with a direct ophthalmoscope with an integrated fixation target, which is the most widely used method to determine the fixation position on the retina [4]. This is of clinical importance because the degree of eccentricity affects the visual acuity [5, 6]. Amblyopes with an eccentric fixation have lower contrast sensitivity and more severe visual acuity loss in comparison with other types of strabismic and/or anisometropic amblyopia with central fixation [7]. Moreover, the presence of eccentric fixation is a factor that negatively influences success in therapy [8–10]. Thus, it is important to investigate specifically this group and its treatment efficiency.

The pathogenesis of eccentric fixation is not yet fully understood. According to Cüppers and his anomalous correspondence theory, there may be a shift of the principal visual direction from the foveola to a different retinal area [3, 11]. The discovery of eccentric fixation was followed by the development of new treatment options like pleoptics [12, 13], red filters [14] and inverse occlusion (occlusion of the amblyopic eye) [15]. Haidinger’s brushes were introduced to detect the fixation locus and train the amblyopic eye to use its fovea for fixation. These treatment options, however, did not show superiority over conventional occlusion treatment [16–18]. Moreover, pleoptic treatment is time consuming, economically costly and needs specially trained staff [13]. Therefore, refractive correction and occlusion of the fellow eye (direct occlusion) are still the mainstay treatment [19].

It is known that amblyopia treatment is most effective during the sensitive developmental period up to 7 years of age and as soon as possible after the onset of amblyopia [2, 20]. Studies also showed some therapy success in older children, but it is of great variability [20–24]. Nevertheless, even when treatment is initiated early and adherence to the therapy is good, up to 45% of amblyopic children retain residual amblyopia [25], and eccentric fixation may be a factor. Regardless of the fact that the presence of eccentric fixation is a factor that negatively influences success in therapy [8, 9], fixation is rarely addressed in recent studies. Additionally, the objective measurement of adherence with occlusion has only been possible in the last two decades [26–29]. Thus, it has not yet been adequately evaluated to which extent the poorer outcome in amblyopes with eccentric fixation is due to a lower adherence. This uncertainty leads to a lack of standardization of the dosage and age limits of the occlusion regimens in this particular group.

Recent studies have begun to explore the dose-response relationship [25, 30, 31] and efficiency of occlusion therapy using electronically measured occlusion rates [20], while considering important factors like therapy adherence and also refractive adaptation phase [32–34]. Although it is known that amblyopic eyes with eccentric fixation have a poor visual outcome [9], until now, there are no prospective studies that provide systematically collected quantitative data of the dose response and treatment efficiency focused on this particular group of patients.

This pilot study is the first to examine objective dose rate, dose-response relationship and efficiency of amblyopia treatment in patients of a large age span with strabismic and combined amblyopia and eccentric fixation. Our aim was to explore the relationship between received occlusion hours and visual acuity as a function of age, therapy duration and change of fixation over a large time span.

## Methods

### Study design and patient inclusion

Children with strabismic and combined amblyopia and eccentric fixation between the age of 3 and 16 years were included in this two-centre prospective pilot study in the University Hospitals of Frankfurt and Tübingen. Patients were recruited from the paediatric ophthalmology departments of the two hospitals, as well as from ophthalmologists’ offices. Before study entry, all patients had full ophthalmic and orthoptic assessment, including fundoscopy, cycloplegic retinoscopy and assessment of the fixation of both eyes using a direct ophthalmoscope. When necessary, patients received a prescription for spectacles with maximum subtraction of 0.5 dioptre of the sphere from the cycloplegic retinoscopy value. Inclusion criteria were visual acuity difference of at least 0.2 logMAR (logarithm of the minimum angle of resolution) after 3 months of refractive adaptation, eccentric fixation of the amblyopic eye, no occlusion therapy at least 1 year prior to enrolment, no deprivation amblyopia and no other ocular diseases or neurological disorders. Criteria for strabismic amblyopia were existence of heterotropia when fixating at distance and/or near or a history of strabismus surgery. If there was a difference of ≥ 1 dioptre in the spherical equivalent or ≥ 1.5 dioptre in astigmatism between both eyes, the amblyopia was classified as combined. Prior to enrolment in the study, parents and patients older than 7 years declared their written informed consent and younger patients their assent. The study was administered according to the Declaration of Helsinki of the World Medical Association. The Ethics Committees of the Universities of Frankfurt and Tübingen approved the study protocol before initiation.

Based on the guidelines of the German Ophthalmological Society (DOG) for amblyopia with eccentric fixation, preschool children < 7 years were prescribed 12 h/day occlusion for as many days as their age followed by a day pause [35]. School children ≥ 6 years were prescribed 6 h/day occlusion on the weekdays and 12 h/day on weekends, as due to poor visual acuity of the amblyopic eye, occlusion was not feasible during school hours. Alternative therapies in addition to occlusion of the fellow eye were not prescribed while participating in the study. Follow-up examinations were performed every 4 weeks in the first 4 months, then at the sixth, ninth and twelfth month. The extended time span of 12 months was chosen due to the eccentric fixation of the amblyopic eye and the large age span also including older children [31, 36]. Visual acuity test and orthoptic assessment, including measuring the angle of squint (using cover/uncover and alternate cover test with prisms), examining binocular vision and assessing fixation of both eyes were executed on every follow-up examination.

Our therapeutic goal was a crowded visual acuity in the normal range for the respective age and/or an interocular visual acuity difference of ≤ 0.1 logMAR at the end of the study. We also aimed for a shift of the fixation locus to the foveola. Patients who reached this therapeutic goal earlier were then prescribed 6 h/day occlusion.

### Visual acuity

Near visual acuity was measured using log-scaled uncrowded and crowded Landolt ring charts at a constant optotype distance of 2.6 arcminute (LC_2.6_, Oculus®). Crowded logarithmic Lea Symbols (CLS, Lea-Test Ltd® by Precision Vision®) were used for children under 4 years of age, who could not be tested successfully while presented a Landolt ring chart. The charts were always presented at exactly 0.4 m distance during the acuity testing (two examiners present). Considering the different inter-optotype distance in both crowded charts, an adjustment was needed prior to the statistical visual acuity evaluation (LC_2.6_ exceeds CLS by 0.2 log units [37]). The chart used at the first examination was used throughout the entire study period.

### Fixation assessment

Fixation assessment was undertaken using a direct ophthalmoscope by two experienced examiners who agreed on the fixation locus. They were both blinded to the actual occlusion hours and previous locus of fixation. We executed the examination in low-intensity illumination of the ophthalmoscope in a darkened room as it is reported that the increased illumination during examination affects the fixation stability [38]. A star was projected as a fixation object on the retina, and its position in relation to the foveolar reflex showed the fixation locus [1, 4, 11]. Because eccentric fixation is rarely stable, we observed the fixation pattern over a period of 20–30 s and chose the most frequently used position on the retina or the midpoint of the range. We diagnosed eccentric fixation based on the fixation locus on the retina and classified the loci in 4 groups: foveolar, parafoveolar (< 2.5°), parafoveal (≥ 2.5°to ≤ 5°), peripheral (> 5°) [39].

### Objective monitoring of occlusion

**Fig. 1:**
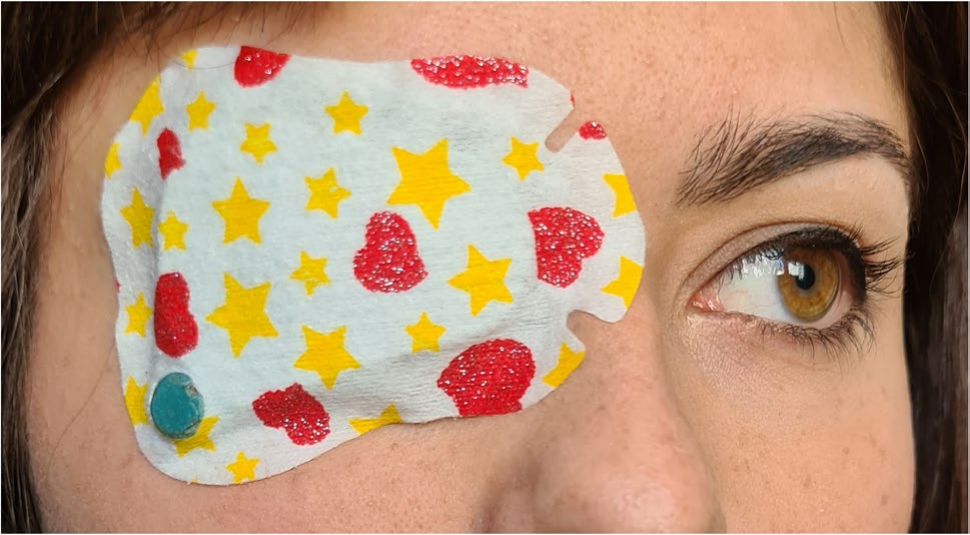
Occlusion patch with a TheraMon® microsensor (9 × 13 × 4.5 mm) attached to its inside and visible through a hole made at the lateral inferior margin of the patch. The microsensor measures the environment temperature every 15 min, has a memory capacity of 100 days and a battery life of 2 years

The wearing time of occlusion patches was continuously recorded using a TheraMon® microsensor (Handelsagentur Gschladt, Hargelsberg, Austria) that was attached to the inside of the patch as seen in Fig. 1. The recordings have been shown to be reliable in previous studies [40, 41]. The temperature and time values from the TheraMon® Software were processed with Visual Basics for Applications in Excel (Microsoft® Excel for Office 365). The mean daily duration of occlusion during 12 months was calculated (dose rate). Wearing time diaries were kept by the parents to compensate for a potential data gap in case of loss of the microsensor. Previous studies have shown that there is a good accordance with occlusion diaries when patients and parents were aware of the monitoring of the therapy, even when adherence with therapy was low [28, 42].

### Outcome measures

Our outcome measures were evaluation of visual function: amblyopic eye visual acuity in logMAR, acuity gain, interocular visual acuity difference (IOVAD), proportion of deficit corrected; adherence based on objective monitoring of occlusion; and fixation pattern of the amblyopic eye. We calculated the proportion of deficit corrected with the formula “(Vai − Vae) / (Vai − Vnae)”, in which Vai is the initial visual acuity of the amblyopic eye, Vae the end visual acuity of the amblyopic eye and Vnae the end visual acuity of the non-amblyopic eye [9]. We explored treatment efficiency and dose-response relationship for amblyopia with eccentric fixation. Treatment efficiency expresses the acuity gain in log units per 100 h of patching and was calculated with the formula “acuity gain/cumulated occlusion hours × 100”. Dose-response relationship was calculated with the formula “cumulated occlusion hours/acuity gain × 0.1”. Unlike dose-response relationship, treatment efficiency can also account for patients with no acuity gain, which is especially useful when older patients and patients with eccentric fixation are included [20].

### Statistical analysis

Relations between variables were examined by Spearman’s rank correlation using Edgeworth approximation. Friedman test with Conover post hoc analysis was applied for comparison of data at different times: study entry and 1/2/3/4/6/9/12 months of occlusion therapy. To statistically analyse differences between different groups of patients, the exact Mann–Whitney *U* test was used. Multiple regression with backward elimination was applied to study influencing factors for visual acuity gain and IOVAD-reduction. *p* values < 0.05 were considered statistically significant. Statistics software BiAS 11.10 was used (© epsilon publishing 1989–2019 Dr. H. Ackermann, Goethe-Universität Frankfurt).

## Results

### Study participants

A total of 17 patients were recruited in this study. Four participants dropped out before the end of the study. One child aged 4.6 years refused to wear the occlusion patch. This child was excluded from the study after 3 months and was prescribed atropine eye drops thereafter. While occluding 2.9 h/day during the first 2 weeks and 0 h/day thereafter, the visual acuity of this child did not improve during 2 months despite its young age (see Fig. 3 a and b, marked with a star). The other three children (age 8.4, 9.2 and 12.8 years) failed to attend the follow-up examinations. One child aged 10.3 years was not prescribed the necessary occlusion hours and thus was excluded due to violation of the study protocol. Therefore, 12 patients between 2.9 and 12.4 years (mean age 6.5 ± 3.4 years, strabismus *n* = 6, combined amblyopia *n* = 6) with a total of 96 visits (8 visits per patient) were included in the following analyses. Their characteristics at study initiation as well as visual acuity at study end and mean duration of occlusion (dose rate) are summarized in Table 1.

**Table 1:** Characteristics of the patients at study initiation including final visual acuity and mean daily duration of occlusion during 12 months (dose rate)

Patient	Age (years)	Type of amblyopia	Fixation	Angle of squint, cover/uncover test with prism (prism dioptres)		Eye	Refraction (glasses)	Visual acuity crowded near (logMAR)		Dose rate (h/day)	History
								initial	final		
				near	distance			AE NAE	AE NAE		
1	2.9	Strab	Peripheral	+ 25	+ 25	LE^a^	+ 5.5 sph	1.7	0.5	6.8	Untreated
						RE	+ 5 sph	0.7	0.4		
2	3.4	Comb	Peripheral	+ 20	± 0	LE^a^	+ 6.75/ − 0.75/49°	2.0	0.7	6.7	Untreated
						RE	+ 3/ − 0.5/2°	0.8	0.4		
3	3.5	Comb	Parafoveal	+ 8^b^	+ 4^b^	RE^a^	− 4.5 sph	1.3	0.4	5.4	Untreated
						LE	− 0.5/ − 0.5/0°	0.5	0.3		
4	3.8	Strab	Peripheral	+ 60	+ 60	RE^a^	+ 2.75/ − 0.5/180°	2.1	0.4	2.9	Untreated
						LE	+ 2.25/ − 0.5/180°	0.5	0.2		
5	3.9	Strab	Parafoveolar	+ 5	+ 5	LE^a^	+ 3.5/ − 1.5/10°	1.2	0.2	6.0	Untreated
						RE	+ 2.5/ − 1/170°	0.5	0.2		
6	4.6	Strab	Peripheral	+ 2	+ 2	RE^a^	+ 7.5/ − 2.75/175°	1.1	0.5	9.7	Untreated
						LE	+ 6.25/ − 2/12°	0.3	0.3		
7	6.5	Strab	Parafoveolar	+ 20	+ 8	LE^a^	+ 1.5/ − 0.5/180°	1.2	0.6	4.9	Pretreated (occlusion at age 5 for 1 year, 5–6 h/day)
						RE	+ 0.75 sph	0	− 0.1		
8	8.1	Strab	Parafoveolar	+ 3	+ 3	LE^a^	+ 8/ − 1.5/35°	1.2	0.8	3.2	Untreated
						RE	+ 7/ − 1/175°	0.2	0		
9	8.4	Comb	Parafoveolar	+ 5	+ 2	LE^a^	+ 1.5/ − 2.5/170°	0.9	0.4	5.7	Untreated
						RE	+ 1/ − 1/180°	0.4	0.2		
10	8.5	Comb	Peripheral	+ 16 + VD 10	+ 8 + VD 10	RE^a^	+ 3/ − 1.5/150°	1.7	1.7	4.1	Untreated (strabismus surgery at age 8)
						LE	+ 1.5/ − 1/165°	− 0.1	− 0.1		
11	12.3	Comb	Parafoveal	− 8^b^	− 6^b^	RE^a^	+ 4/ − 2/20°	1.3	1.2	1.8	Untreated
						LE	+ 1/ − 0.5/170°	− 0.1	− 0.1		
12	12.4	Comb	Parafoveolar	+ 2	+ 3	RE^a^	+ 6 sph	0.9	0.8	0.7	Pretreated (occlusion at age 9 for 1 year, 4 h/day, strabismus surgery at age 10)
						LE	none	− 0.1	0.1		

*Strab* strabismus, *Comb* combined, *AE* amblyopic eye, *NAE* non-amblyopic eye, *VD* vertical deviation, *h/day* hours per day

^a^Amblyopic eye

^b^Angle of squint at the second examination when an angle of squint at the first examination was 0 prism dioptres but greater every time after that

We present results on near crowded acuity. Mean initial acuity of the amblyopic eyes was 1.4 ± 0.4 logMAR (range 0.9–2.0) and of the fellow eyes 0.3 ± 0.3 logMAR (range − 0.1–0.8). There was a tendency towards a better initial acuity of the amblyopic eye with increasing age, but it did not reach significance (Spearman’s rank correlation, rho =  − 0.56, *p* = 0.06). Fellow eyes showed significantly better visual acuity with increasing age (Spearman’s rank correlation, rho =  − 0.93, *p* ≤ 0.001). Mean initial interocular visual acuity difference (IOVAD, visual acuity difference between amblyopic and fellow eye) was 1.1 ± 0.4 log units (range 0.5–1.8), and there was no significant correlation between IOVAD and age (Spearman’s rank correlation, rho = 0.12, *p* = 0.69).

All categories of eccentric fixation were found in our study participants. Parafoveolar (*n* = 5) and parafoveal (*n* = 2) fixation loci were distributed evenly among all ages, peripheral fixation (*n* = 5) was predominant in patients ≤ 4 years.

Patients and their parents were specifically asked about adverse effects like diplopia and any other complaints. None of the children developed double vision. One child (3.9 years old) developed an allergy to the occlusion patch and had to switch to occluding with an eye patch cover for glasses after 2 months. Due to very good agreement between protocol and objectively measured occlusion, protocol data was used thereafter.

### Prescribed and electronically recorded occlusion

Median prescribed occlusion of the whole group was 7.7 h/day (range 6.6–9.9). Median prescribed occlusion in the preschool group was 8.1 h/day (range 6.6–9.9) and in the school group during the whole study period 7.7 h/day. The difference between the two groups after 12 months was not significant (Mann–Whitney *U* test, *p* = 0.39). There was however a slight difference in the first 4 months (preschool: median 9 h/day, school: median 7.7 h/day, Mann–Whitney *U* test, *p* = 0.02).

**Fig. 2:**
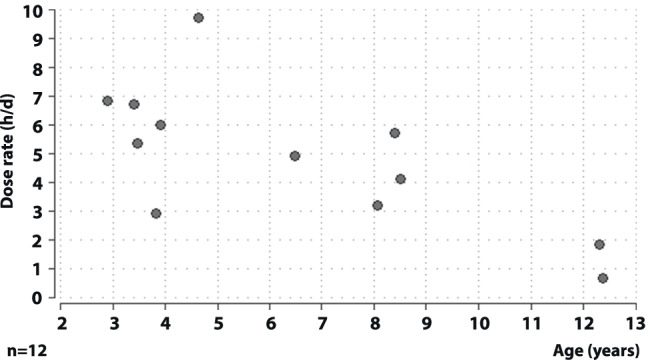
Correlation between dose rate (mean occlusion in hours per day) during 12 months and age

Median recorded occlusion in the whole group during the 12 months of treatment was 5.2 h/day (range 0.7–9.7). Median adherence with prescribed occlusion in the first 4 months was 81% in the preschool group (6.8 h/day) and 60% in the school group (4.6 h/day) and during the 12 months 84% (6.4 h/day) and 53% (3.7 h/day), respectively. As shown in Fig. 2, occlusion decreased with age. There was a significant correlation between accomplished occlusion hours and age (Spearman’s rank correlation, rho =  − 0.67, *p* = 0.02), but not between occlusion hours and initial acuity (Spearman’s rank correlation, rho = 0.05, *p* = 0.89). There was no significant difference between different time spans of the study period in preschool children (Friedman test, *p* = 0.58). School children, on the other hand, occluded significantly less after the 6th month despite equal prescription over the whole period (Friedman test, *p* = 0.02).

### Visual acuity

**Fig. 3:**
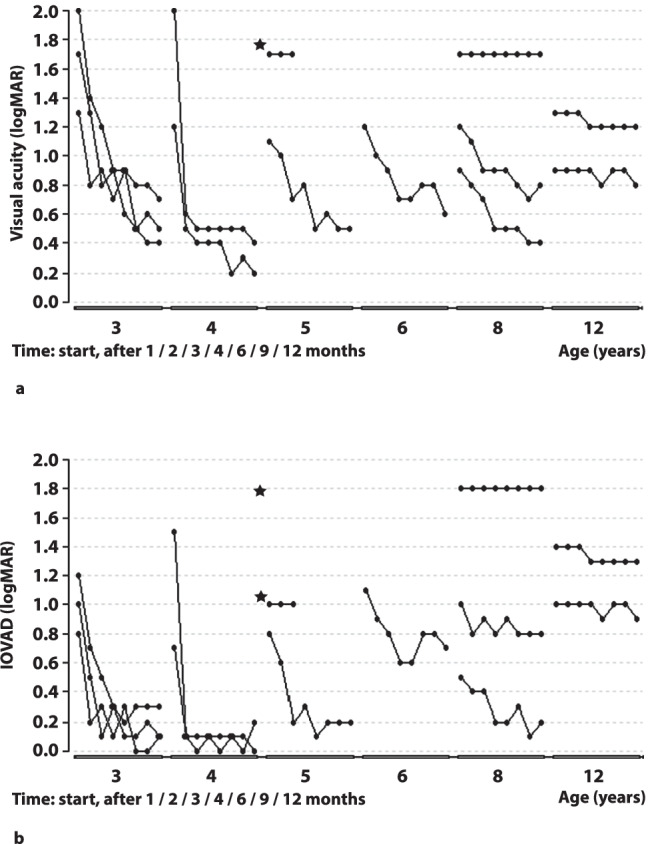
Results of each patient are shown at start and after 1/2/3/4/6/9/12 months and are grouped by age. **a** Near crowded visual acuity in the amblyopic eyes in logMAR. **b** Near crowded IOVAD in logMAR. The child marked with an asterisk was excluded from the study after 2 months because of occlusion patch intolerance. Despite young age, after very few hours of patching in the first 2 weeks and none thereafter, there was neither gain in visual acuity, nor reduction in IOVAD

Most marked acuity gain in the amblyopic eyes was achieved after 3 months with a median gain of 0.5 log units (range 0–1.5). Over the last 6 months of treatment, the visual acuity of 7 patients (5 of them < 7 years) continued to improve slightly (median 0.1, range 0.1–0.2). At the end of the study, three patients (all ≥ 8 years) did not achieve clinically relevant acuity gain of at least 0.2 log units. Two of them were 12 years old with low dose rate (< 2 h/day), and one was 8.5 years old with a fairly good dose rate of 4.1 h/day, but with a far peripheral fixation near the optic disc. All other patients had acuity gain of at least 0.4 log units after 12 months (whole group median 0.6 log units, range 0–1.6). The maximum visual acuity achieved by our patients with residual eccentric fixation was 0.6 logMAR tested with crowded optotypes and − 0.1 logMAR tested with uncrowded optotypes. There was no significant correlation between initial visual acuity and acuity gain at the end of the study (Spearman’s rank correlation, rho = 0.49, *p* = 0.11). Figure 3a shows the visual acuity progress of each patient as a function of time (grouped by age). Spearman’s rank correlation revealed a strong correlation between age and acuity gain at the end of study (rho =  − 0.91, *p* ≤ 0.001). A multiple regression analysis with backward elimination showed that both age (*p* = 0.0002) and dose rate (*p* = 0.046) were significant influencing factors for visual acuity gain at the end of study. After 12 months, patients < 7 years showed an improvement in visual acuity of median 1.0 log units (range 0.6–1.6) and patients ≥ 7 years: 0.1 log units (range 0–0.5).

### IOVAD

We additionally calculated the IOVAD because unlike visual acuity in the amblyopic eyes, this parameter considers the physiological improvement in visual acuity in developing young children, which especially applies to crowded acuity [43].

**Fig. 4:**
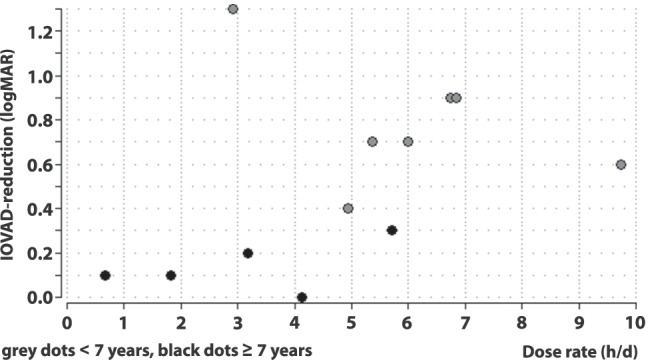
IOVAD-reduction in logMAR after 12 months in correlation to occlusion in hours per day during 12 months. Grey dots represent children < 7 years and black dots ≥ 7 years

Figure 3b shows the change in IOVAD of each patient as a function of time (grouped by age). There was a lower but still rapid and clear decrease in IOVAD in younger children compared to visual acuity gain in the amblyopic eye. It underlined the more distinct therapy success in younger patients even when the physiological increase in acuity was being taken into consideration. Spearman’s rank correlation showed a very strong correlation between age and IOVAD-reduction after 12 months (rho =  − 0.92, *p* ≤ 0.001). There was no significant correlation between initial IOVAD and IOVAD reduction after 12 months (Spearman’s rank correlation, rho =  − 0.12, *p* = 0.69). At the end of the study, the median residual IOVAD in the whole group was 0.3 log units (range 0–1.8). Patients < 7 years of age showed a residual median IOVAD of 0.2 log units (range 0–0.7) and ≥ 7 years of 0.9 log units (range 0.2–1.8). Five patients (< 5 years old at therapy initiation) reached an IOVAD of less than 0.2 log units at some point during the study, fulfilling the criterion for cured amblyopia. All patients but one (3.5 years, occlusion 5.4 h/day) reached their minimal IOVAD within 4 months of therapy (median 3 months, range 1–6 months). After achieving central fixation (median after 3 months), there was no further median reduction in the IOVAD (range − 0.1–0.1). Median reduction in IOVAD before achieving central fixation was 0.6 log units (range 0–1.4). The relationship between IOVAD-reduction and mean dose rate (differentiated between children younger and older than 7 years) is shown in Fig. 4. In the overlapping area with similar dose rates of younger and older children, the younger children had a noticeably higher IOVAD-reduction (Mann–Whitney *U* test, *p* = 0.003). A multiple regression analysis with backward elimination showed that age was a significant influencing factor for IOVAD-reduction at the end of the study (*p* = 0.0005) while higher dose rate showed a tendency for a better IOVAD-reduction (*p* = 0.09).

As stated above, 7 patients still showed some improvement in amblyopic eye visual acuity over the last 6 months of treatment. However, no patient showed a consistent reduction in IOVAD during that time. Five patients showed a fluctuation of 1 line (range 3.9–12.4 years). Moreover, visual acuity gain and IOVAD-reduction showed similar results until the 4th month but ≥ 0.2 log units difference after the 6th month.

Despite clear improvement in visual acuity during treatment, both final visual acuity and IOVAD assessed with crowded Landolt rings were still far from normal except in the youngest patients (see Fig. 3a and b). However, it is worth mentioning that when measured with an uncrowded Landolt ring chart, both visual acuity and IOVAD were much more favourable, resulting in near normal median amblyopic eye acuity for the age. This is shown in Table 2, where we compared visual acuity and IOVAD of the nine patients in whom acuity could be measured with both crowded and uncrowded optotypes.

**Table 2:** Crowded and uncrowded visual acuity of the amblyopic eye and IOVAD at initiation and end of the study. Median logMAR, range in brackets. Landolt ring chart with an optotype spacing of 35 arcminutes was used to measure uncrowded acuity and 2.6 arcminutes for crowded acuity (*n* = 9, age range 3.5–12.4 years)

	Visual acuity at initiation	Visual acuity at end	IOVAD at initiation	IOVAD at end
Crowded	1.2 (0.9–2.1)	0.6 (0.4–1.7)	1.0 (0.5–1.8)	0.7 (0.1–1.8)
Uncrowded	0.8 (0.6–2.1)	0.1 (− 0.1–1.1)	0.8 (0.6–2.1)	0.2 (0–1.2)

### Proportion of deficit corrected

**Fig. 5:**
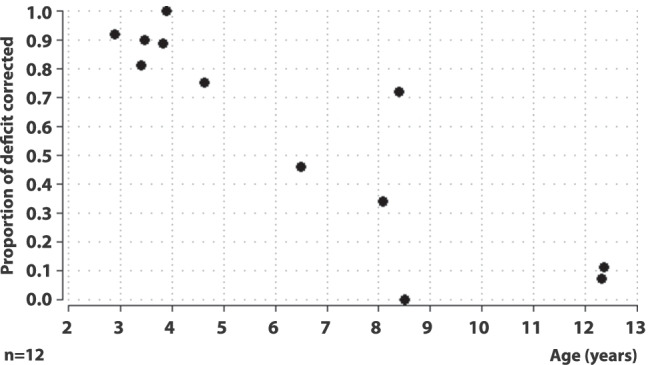
Correlation between proportion of deficit corrected and age after 12 months of occlusion treatment

The relationship between proportion of deficit corrected and age is shown in Fig. 5. The proportion of deficit corrected was 0.75–1.0 in all six children younger than 5 years (50% of the study population), 0.5– < 0.75 in one child aged 8.4 years (8%), 0.25 < 0.5 in two children aged 6.5 and 8.1 years (17%) and < 0.25 in three children aged 8.5–12.4 years (25%). In three children aged 2.9–3.9 years, the proportion of deficit corrected was 0.9–1.0. There was a significant correlation between proportion of deficit corrected and age (Spearman’s rank correlation, rho =  − 0.86, *p* = 0.0006).

### Treatment efficiency

Efficiency data is summarized in Table 3. Efficiency calculation showed an acuity gain of approximately one line from 100 h of patching in the first 2 months and half a line after 6 months reflecting the steeper initial improvement in visual acuity especially in children < 7 years. There was a significant correlation between treatment efficiency and age at the end of the study (Spearman’s rank correlation, rho =  − 0.71, *p* = 0.01). Patients ≥ 7 years showed in median zero efficiency during the first 2 months and an increase in efficiency thereafter. When using uncrowded optotype visual acuity gain to calculate efficiency in those patients, efficiency was overall higher and decreased after the 6th month. This suggests that improvement in crowded acuity in older children occurs later than in uncrowded acuity.

**Table 3:** Median efficiency in log units acuity gain per 100 h occlusion, *n* = 12, < 7 years *n* = 7, ≥ 7 years *n* = 5. In children ≥ 7 years, also efficiency based on uncrowded visual acuity data is presented

Efficiency	1 month	0–2 months	0–3 months	0–4 months	0–6 months	0–12 months
Whole group	0.113	0.102	0.090	0.062	0.053	0.039
< 7 years	0.224	0.155	0.117	0.086	0.081	0.046
≥ 7 years	0	0	0.026	0.044	0.016	0.024
≥ 7 years (uncrowded optotypes)	0.043	0.049	0.050	0.045	0.033	0.028

### Dose response relationship

The dose response data from patients < 7 years is summarized in Table 4. It reveals that dose response to treatment gets more unfavourable with time, reflecting the more pronounced improvement during the initial phase of treatment. A total of 232.6 h of occlusion during the first 4 months was needed to gain 0.2 logMAR acuity (clinically significant acuity gain). Data for patients ≥ 7 years and for the whole group could not be calculated because up to 3 patients (all > 8 years) would have to be excluded due to the lack of acuity gain at different times (and the consequent division by zero).

**Table 4:** Median dose response in hours per 0.1 log units gain for patients < 7 years, range in brackets, *n* = 7

Dose response	1 month	0–2 months	0–3 months	0–4 months	0–6 months	0–12 months
< 7 years	44.6 (9.8–303)	64.6 (21.1–131.8)	85.2 (29.6–268.1)	116.3 (34.5–175.8)	123.5 (42.6–332.8)	215.5 (65.9–561.8)

### Fixation

Foveolar fixation was reached by nine patients after median 3 months (range 1–4) and by all patients < 6 years. Three patients could not gain central fixation in the study period (all ≥ 6 years, median occlusion 4.1 h/day). Two of them did not show improvement towards foveal fixation and in visual acuity. One of them, aged 6.5 years, showed a slight shift of fixation towards the foveola and IOVAD-reduction of 0.5 logMAR but still had an IOVAD of 0.7 logMAR at the end of the study after mean occlusion of 4.9 h/day. Spearman’s rank correlation did not show a significant correlation between age and time to achieving foveolar fixation (rho = 0.46, *p* = 0.21). However, children who did not reach central fixation were not included in this calculation.

**Fig. 6:**
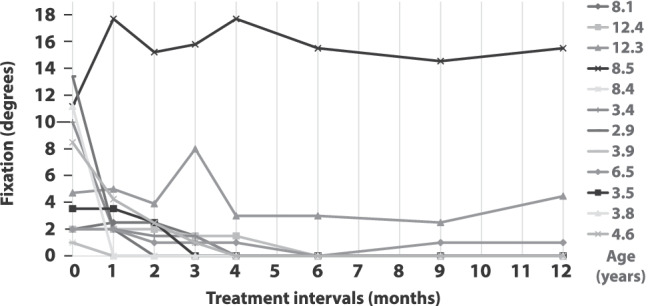
Change in retinal fixation in degree of every patient during the study period, including clinical categories of fixation pattern. Age of every patient is shown on the right

Figure 6 shows the individual locus of fixation of each patient on every visit. Four out of five patients (80%) with peripheral fixation achieved a central fixation (all < 5 years, median occlusion 6.8 h/day), and one stayed peripheral during the whole study period (8.5 years, mean occlusion 4.1 h/day). Only the younger one of two children with parafoveal fixation (3.5 years, occlusion 5.4 h/day and 12.3 years, occlusion 1.8 h/day) reached central fixation. Four out of five patients (80%) with parafoveolar fixation gained central fixation (three of them > 8 years, age range 3.9–12.3 years, median occlusion 4.5 h/day). One patient aged 6.5 years with parafoveal fixation could not achieve central fixation despite 4.9 h/day of mean occlusion.

## Discussion

Our prospective pilot study was the first to explore the relationship between electronically monitored occlusion and visual function specifically in amblyopic patients with eccentric fixation while studying the occurring fixation shift. Examining a wide age span, beginning with the traditional age but also extending past it, allowed us to investigate the relationship between age and treatment efficiency in this aetiology. The rarity of this patient group explains the relatively small sample size. Nevertheless, the relationship between occlusion hours and visual acuity in relation to age and treatment duration could be investigated. Despite occasional moderate improvement even in older children, the best therapy response was found for patients < 4 years.

### Visual acuity and IOVAD

In our study, young children were also included, which explains the wide crowded visual acuity range with relatively high logMAR values in the fellow eye, but also distinctly higher values in the amblyopic eye. The proportion of amblyopia severity was comparable among younger and older patients. While there was a tendency towards better initial acuity of the amblyopic eye with increasing age, there was no significant correlation between initial IOVAD and age. IOVAD is thus a useful parameter when comparing younger and older patients over a long period of time since it considers the physiological acuity gain in young children.

Our study yielded a significant correlation between acuity gain and age (rho =  − 0.91, *p* ≤ 0.001). The greater treatment success in younger children in our study could be emphasized through the still higher decrease in IOVAD when compared to older patients (Fig. 3b). Seventy-five per cent of our patients (9 out of 12, all ≤ 8 years) had clinically relevant acuity gain of at least 0.2 log units. Moreover, using a multiple regression analysis, it could be shown that visual acuity gain and IOVAD were influenced much stronger by age than by dose rate. Similarly, in the PEDIG study, patients with severe amblyopia younger than 5 years achieved greater improvement than older patients [10]. This was also observed in the ROTAS [44]: patients under 4 years of age showed significantly more improvement in the proportion of deficit corrected even at low dose rates.

Few corresponding studies are available for appraisal of our data. In our study, patients < 7 years (mean age 4.1) showed an improvement in visual acuity of median 1.0 log units (range 0.6–1.6) after 12 months of occlusion. In comparison, other studies which did not explicitly examine amblyopes with eccentric fixation showed less acuity gain in this age range. In the PEDIG study [10] on severe amblyopia (average visual acuity gain 0.5 logMAR, age 3–7 years), no objective monitoring of the occlusion hours was undertaken and therefore, it is uncertain whether the patients actually occluded full-time. Whereas in our study, adherence with prescribed occlusion in this age group was 84% (6.4 h/day). Children aged 3–8 years gained in the MOTAS [25] 0.35 log units (0.0–1.2) visual acuity and in ROTAS [44] 0.24 log units (0.19–0.29), while occluding 2.8 h/day and 6.2 h/day, respectively. An important factor to consider is that our patients with eccentric fixation had a much more severe amblyopia than the latter, thus allowing for much higher acuity gain.

In the first 4 months of occlusion treatment, our patients ≥ 7 years (mean 9.9 years) had median visual acuity improvement of 0.1 log units and after that 0.0 log units (range for the whole treatment period 0–0.5). Similar results were presented in the study by Kracht et al. [36], which also examined older children over the extended period of 12 months (0.2 log units over the first 4 months, 0.1 log units after that, range − 0.1–0.6). Both studies showed that in children older than 7 years, the maximum acuity gain occurred within the first 4 months and after that, there was in median no clinically significant crowded acuity gain. It should be noted that when examining amblyopes with eccentric fixation with crowded optotypes, visual acuity gain is less distinct, especially in those ≥ 7 years. However, in the Kracht et al. study, there were 3 patients who gained 0.2 logMAR acuity even after the 4th month of occlusion whereas in our study, there were no such patients. In the study by Fronius et al. [20] (5–16 years of age, mean 9.2), median gain in crowded acuity after 4 months was 0.3 log units (range 0–1.4), while occlusion was on average 1 h less than in our study (4.2 h/day versus 5.2 h/day). In summary, our patients ≥ 7 years showed less gain than in the studies mentioned above, which could be explained by the fact that most children from those studies had central fixation.

The highest acuity gain and reduction in IOVAD occurred during the first 3 months, and there was no clinically significant decrease in IOVAD over the last 6 months of our study. Interestingly, there was no further mean reduction in the IOVAD after achieving central fixation. Nevertheless, this correlation does not necessarily imply causation, because the maximum IOVAD-reduction and central fixation are usually both achieved during the first months of treatment. The median IOVAD at the end of our study was 0.3 log units (range 0–1.8) which is in accordance with the subsample of patients with eccentric fixation from the MOTAS [9] with median IOVAD of 0.36 log units (range 0–0.98). Patients ≥ 7 years of age showed median IOVAD at the end of our study of 0.9 log units (range 0.2–1.8), which is higher than the residual IOVAD of patients with severe amblyopia from the PEDIG-trial [22] with 0.51 logMAR. This could be due to the eccentric fixation of our patients, as patients with eccentric fixation were shown to have greater residual amblyopia than patients with central fixation [9].

### Occlusion and adherence

Our data showed how much occlusion per day children with severe amblyopia and eccentric fixation can incorporate in their everyday lives. Because school children could not occlude full-time on weekdays due to school visits, there was a significant difference in prescribed occlusion between preschool and school children in the first 4 months. Yet, school children showed a lower adherence with prescribed occlusion (53% versus 84% in preschool children), and despite equal prescription over the 12 months, occlusion hours in school children dropped significantly after the 6th month.

When comparing our results in the first 4 months with the patients from ROTAS [44], who were also prescribed full-time occlusion, our patients were more adherent to the therapy (71% versus 52%). Our results were comparable with two studies from Germany by Fronius et al. [20] (78% adherence during 4 months) and Kracht et al. [36] (80% in the first 4 months and 72% during 4–12 months), which however had lower prescription (5.4 h/day and 6 h/day). The first two studies mentioned above [20, 44] showed no significant relationship between adherence and age. This is comprehensible in the ROTAS due to the smaller age span including only younger children (3–8 years) and in Fronius et al. due to the lower prescription (5.4 h/day).

### Dose response and efficiency

Dose response and treatment efficiency are approaches to defining effectiveness of occlusion treatment and to concluding on plasticity of the visual system. Dose-response relationship expresses the occlusion hours needed to gain 0.1 log unit of acuity, thus not calculable if acuity gain is zero. All patients can be included in efficiency calculations regardless of their acuity gain, which is helpful in reducing the bias when excluding patients for dose-response calculations. Nevertheless, both calculations have their limitations when it comes to extreme occlusion hours and clinically insignificant fluctuations of 0.1 log units in visual acuity. Higher treatment efficiency was measured when patients occluded very little but had a fluctuation of 0.1 log units. Lower efficiency was measured when patients were highly adherent and occluded great amounts of hours, possibly because a treatment-efficiency ceiling per day exists.

Our data showed significant correlation between age and treatment efficiency which is in accordance with the study by Fronius et al. [20]. Efficiency data of the whole group in both studies was of the same order of magnitude: after 1 month in our study 0.113 (range 0–1.025) and 0.125 (range − 0.08–0.92) in the study by Fronius et al. [20], after 4 months 0.062 (range 0–0.290) and 0.05 (range 0–0.39), respectively. Important to note is that patients from our study showed similar efficiency as those from Fronius et al. [20] despite being much younger (mean age 6.5 years, range 2.9–12.4 versus mean 9.2 years, range 5.4–15.8) probably due to the eccentric fixation of the amblyopic eyes of our patients. Children < 7 years of age needed less occlusion hours than children > 7 years to achieve 0.1 log unit acuity gain. When compared, our data from patients < 7 years (mean age 4.1) showed a more unfavourable dose-response relationship than patients aged 4 years from the MOTAS [30]. MOTAS patients needed 170 h of occlusion for 2 lines of acuity gain and our patients 233 h during the first 4 months. The lower response to treatment could be because of the eccentric fixation of all our patients, while in MOTAS, only 29% of patients had eccentric fixation [30, 9]. 

### Fixation

In our study, we examined for the first time the shift in fixation in the amblyopic eye while objectively monitoring the adherence to occlusion treatment. For the assessment of fixation pattern in addition to fixation locus, other parameters such as zero retinomotor point (the point of reflex fixation) and primary visual direction could also be taken into consideration [45]. Due to the age range of our patients, including children from 2.9 years, it was not feasible to examine the primary visual direction. It is known that assessment of fixation using a direct ophthalmoscope is subjective and associated with some uncertainty; still, it has been clinically used and established for a long time. In our study, the fixation locus was assessed by the same two experienced examiners who were both blinded to the previous fixation loci and observed the fixation pattern over a period of time.

The relationship between the magnitude of the visual acuity reduction and the degree of eccentric fixation is a controversial issue. The maximum visual acuity that can be achieved with an existing eccentric fixation is not only depending on the fixation locus but also on the existing sensory inhibition [46].

All patients < 6 years at the start of the occlusion treatment achieved foveolar fixation during the first 4 months regardless of the initial locus of eccentric fixation. Central fixation was reached by all patients in median after 3 months. Similar to our results, the mean time to reach central fixation according to the doctoral thesis of Hillesheim (cited in [39]) was 4 months (with 75% of patients reaching it after 3 months) and according to the study by Gusek-Schneider [47], it was 4.5 months. In Gusek-Schneider`s study, there was no significant correlation between age and time to achieving foveolar fixation, which is in accordance with our data.

Three children (25% of our study population, all ≥ 6 years) did not gain central fixation while occluding median 4.1 h/day. About 40% of the patients from the doctoral thesis of Hillesheim did not reach it either (cited in [39]). Von Noorden examined the therapy success of different fixation loci [15]. In his study, only 27% of the patients with a peripheral fixation achieved central fixation, whereas in our study, their number was 80%. This could be because in our study, all children with a peripheral fixation but one were < 5 years. Children with parafoveal fixation reached in both studies central fixation in 50% of the cases. However, in our study, this group had a small sample size. In the study by von Noorden, 82% of the children with a parafoveolar fixation reached central fixation, which agrees with our results (80%).

## Conclusion

Our study showed for the first time prospective quantitative data on the dramatic decrease in efficiency of occlusion treatment with increasing age in the rare group of amblyopes with eccentric fixation. The visual acuity gain was influenced much stronger by age (*p* = 0.0002) than by the dose rate (*p* = 0.046). Only children < 4 years could achieve near normal visual acuity for their age in their amblyopic eyes and an IOVAD < 0.2 log units while occluding minimum 2.9 h/day. Our data suggest that for children under 4 years of age at the start of the occlusion therapy (good adherence provided), an interocular visual acuity difference of ≤ 0.3 logMAR after 1 year of treatment can be predicted to parents irrespective of the initial locus of fixation. Children < 7 years of age needed less occlusion hours than children > 7 years to achieve 0.1 log unit acuity gain. All children < 6 years achieved central fixation. Moreover, older children were found to be less adherent to the treatment. Especially in the group of eccentric fixators, which requires more intense occlusion, a lower adherence is a negative influencing factor. Therefore, despite possible moderate improvement even in patients > 8 years, there is an urgent need of early diagnosis and intense therapy of this particular group.

The presence of even small degrees of eccentric fixation is a challenge to the therapy success: children with eccentric fixation tended to have lower efficiency than children with foveal fixation. Future studies with objective monitoring of occlusion in larger patient groups are needed in order to establish treatment regimens suitable for children with eccentric fixation according to their age.

## Data Availability

Reported data are available within the article. To request access to the data a data use agreement must be signed by the researcher. All data are to be used for non-commercial purposes only.
